# Microbiota in Gut‐Heart Axis: Metabolites and Mechanisms in Cardiovascular Disease

**DOI:** 10.1002/cph4.70024

**Published:** 2025-06-21

**Authors:** Narendra Kondapalli, Venkatesh Katari, Kesha K. Dalal, Sailaja Paruchuri, Charles K. Thodeti

**Affiliations:** ^1^ The University of Toledo College of Medicine and Life Sciences Toledo Ohio USA

## Abstract

Emerging evidence highlights the pivotal role of gut microbiota in regulating cardiovascular health and disease. The gut microbiota, a diverse community of microorganisms residing in the gastrointestinal tract, interacts with its host through metabolites, immune modulation, and systemic signaling pathways, collectively shaping cardiovascular physiology. Dysbiosis, or an imbalance in gut microbial composition, has been linked to various cardiovascular diseases (CVDs), including hypertension, heart failure and atherosclerosis. Key microbial metabolites such as short‐chain fatty acids (SCFAs), trimethylamine N‐oxide (TMAO) and lipopolysaccharides (LPS) have been implicated in mechanisms involving endothelial, cardiac fibroblast, cardiomyocyte dysfunction, systemic inflammation, and metabolic dysregulation. This review explores the dynamic interplay between the gut and the heart, focusing on: gut microbiota composition and its alterations in CVD; microbial‐derived metabolites and their mechanistic roles in cardiovascular pathophysiology; pathways linking gut dysbiosis to endothelial, cardiac fibroblast and cardiomyocyte dysfunction, inflammation, and immune responses; and therapeutic opportunities targeting the gut‐heart axis, including dietary interventions, prebiotics, probiotics and emerging microbiota‐based strategies. By unraveling these intricate relationships, we aim to provide a comprehensive understanding of how gut microbiota shape CVD pathophysiology and discuss potential avenues for novel therapeutics in precision medicine.

## Graphical abstract

Influence of Dietary and Lifestyle Factors on Gut Microbiota and Cardiovascular Health.

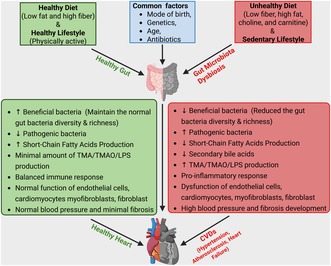

**Abbreviations**
18‐HEPE: 18‐hydroxyeicosapentaenoic acid
β‐MHC: β‐myosin heavy chain
ADP: adenosine diphosphate
ANP: atrial natriuretic factor
BAs: bile acids
BP: blood pressure
CDCA: chenodeoxycholic cid
CHD: coronary heart disease
CKD: chronic kidney disease
CNS: central nervous system
cPLA2a: cytosolic phospholipase A2a
CVDs: cardiovascular diseases
CYP7A1: cholesterol 7a‐hydroxylase
DMB: 3,3‐dimethyl‐1‐butanol
DOCA: deoxycorticosterone acetate
ECCs: enterochromaffin cells
ECM: extracellular matrix
FFAR: free fatty acid receptors
FMT: fecal microbiota transplantation
FXR: Farnesoid X Receptor
GALT: gut‐associated lymphoid tissue
GLP‐1: glucagon‐like peptide‐1
GPCR: G‐protein‐coupled receptors
HF: heart failure
HFpEF: heart failure with preserved ejection fraction
HFrEF: heart failure with reduced ejection fraction
IAA: indole‐3‐acetic acid
IBD: inflammatory bowel disease
ICAM‐1: intercellular adhesion molecule‐1
ImP: imidazole propionate
IMT: intima‐media thickness
IPA: indole‐3‐propionic acid
IS: indoxyl sulfate
LCA: lithocholic acid
LPS: lipopolysaccharides
LXR: Liver X Receptor
mtROS: mitochondrial reactive oxygen
NAFLD: nonalcoholic fatty liver disease
NLRP3: nucleotide‐binding oligomerization domain (NOD)‐like receptor protein 3
NO: nitric oxide
ox‐LDL: oxidized LDL
PAGln: phenylacetyl glutamine
PCS: p‐cresol sulfate
Phe: phenylalanine
PKC: protein kinase C
PRRs: pattern recognition receptors
PXR: Pregnane X Receptor
RAAS: renin‐angiotensin‐aldosterone system
RCT: reverse cholesterol transport
ROS: reactive oxygen species
SCFARs: short‐chain fatty acids receptors
SCFAs: short‐chain fatty acids
SHR: spontaneously hypertensive
SIRT3: sirtuin 3
T2D: type 2 diabetes
TAC: transverse aortic constriction
TGF‐β: transforminggrowth factor‐β
TGR5: Takeda G protein‐coupled Receptor 5
TLR‐4: Toll‐Like receptor 4
TMAO: trimethylamine N‐oxide
Trp: tryptophan
Tyr: tyrosine
VCAM‐1: vascular cell adhesion molecule‐1
VDR: vitamin D receptor
VECs: vascular endothelial cells
VSMCs: vascular smooth muscle cells

## 1 Introduction

The gut microbiota, composed of trillions of microorganisms (bacteria, archaea, viruses, and fungi), is a key regulator of human health, influencing physiological and pathological processes. It symbiotically develops with its host, producing short‐chain fatty acids (SCFAs) like butyrate, acetate, and propionate through the degradation of substrates, nondigestible polysaccharides, including dietary fibers and resistant starches, which support colonocyte energy, immune modulation, and metabolism (Rios‐Covian et al. 2016; Louis and Flint 2009; Correa‐Oliveira et al. 2016; den Besten et al. 2013). Additionally, it synthesizes essential nutrients, vitamin K, and B group vitamins including biotin, cobalamin, folates, nicotinic acid, pantothenic acid, pyridoxine, riboflavin, and thiamine (Hill 1997) and plays some pivotal functions in the development and modulation of the immune system, training immune cells to distinguish between pathogens and beneficial microbes (LeBlanc et al. 2013; Belkaid and Hand 2014). The gut microbiota can maintain epithelial homeostasis to support the development of gut‐associated lymphoid tissue (GALT) and also enhances epithelial cytokine production, which regulates the action of T and B lymphocytes, macrophages, and polymorphs, maintains gut barrier integrity, and prevents systemic inflammation (Belkaid and Hand 2014; Geem et al. 2014; Yoon and Kim 2018; Wu and Wu 2012; Round and Mazmanian 2009; Wells et al. 2017; Jiang and Wu 2022). The microbiota also communicates bidirectionally with the central nervous system (CNS) via different pathways, including endocrine, immune, metabolic, and neuronal pathways (Banfi et al. 2021). In addition, gut metabolites and chemical substances, such as SCFAs and neurotransmitters produced by the gut microbiota directly influence mood, cognition, and behavior (Cryan et al. 2019; Carabotti et al. 2015). The healthy gut microbiota creates a competitive environment by occupying niches and prevents pathogen intestinal colonization through multiple mechanisms, including nutrient competition, and produces small antimicrobial peptides (Horrocks et al. 2023). It regulates glucose and lipid metabolism by influencing insulin sensitivity, bile acid metabolism, and adipose tissue inflammation, playing a role in conditions like obesity and type 2 diabetes (T2D) (Zhao 2013; Kootte et al. 2012). Dysbiosis—imbalances in microbial composition—is linked to various diseases, including inflammatory bowel disease (IBD) and irritable bowel syndrome (IBS) (Franzosa et al. 2019); metabolic disorders like obesity, diabetes, and nonalcoholic fatty liver disease (NAFLD) (Canfora et al. 2019; Tilg and Moschen 2014); immune dysregulation (allergies, asthma, and autoimmune diseases) (Round and Mazmanian 2009; Hooper and Macpherson 2010); and cardiovascular diseases (CVDs) (Wang et al. 2011; Tang et al. 2017).

Recent studies show that evidence of IBD is associated with CVDs such as coronary artery disease, atrial fibrillation, stroke, and heart failure (HF). Mechanisms between IBD and CVDs included atherosclerosis/endothelial dysfunction, dyslipidemia, thrombocytosis, dysbiosis of gut microbiota, and IBD medications (Chen et al. 2022). It has been reported that gut dysbiosis in IBD increases intestinal permeability, allowing bacterial products to enter the bloodstream, which promotes inflammation and endothelial dysfunction, contributing to CVD (Sanchez Cruz et al. 2024). For instance, the alterations in the Firmicutes/Bacteroidetes ratio associated with high blood pressure (BP) and the enrichment in Streptococcus spp.‐ Enterobacteriaceae, including *E. coli* , which is observed in patients with IBD and CVD (Walker et al. 2011; Baumgart et al. 2007; Jie et al. 2017).

The gut and cardiovascular system interact through microbial metabolites, inflammatory pathways, neurohormonal signals, and the autonomic nervous system, forming the gut‐heart axis (Singh et al. 2024). Gut microbiota influences cardiovascular health by producing beneficial metabolites, regulating lipid metabolism, reducing inflammation, and supporting vascular function (Aziz et al. 2024). For instance, SCFAs modulate BP by acting on G‐protein‐coupled receptors (GPR41/43), influencing vascular tone and inflammation (J. L. Pluznick 2017; Du et al. 2024; Kristev et al. 1991). Microbial‐derived trimethylamine (TMA) is converted in the liver to trimethylamine N‐oxide (TMAO), which promotes atherosclerosis by increasing cholesterol deposition and impairing reverse cholesterol transport (RCT) (Wang et al. 2011; Tang, Backhed, et al. 2019). Dysbiosis can increase gut permeability (leaky gut), allowing translocation of microbial products like lipopolysaccharides (LPS), triggering systemic inflammation and endothelial dysfunction, a hallmark of CVDs (Cani et al. 2007). Alternatively, conditions like HF and chronic hypertension can induce intestinal hypoperfusion, ischemia, and congestion, leading to altered microbial diversity and increased gut permeability (Kummen et al. 2018; Sandek et al. 2007). Medications used in CVDs management, such as statins, beta‐blockers, angiotensin‐converting enzyme inhibitors, and platelet aggregation inhibitors, can modulate gut microbiota composition (Tuteja and Ferguson 2019). Restoring gut homeostasis through probiotics, prebiotics, dietary interventions, and fecal microbiota transplantation (FMT) holds promise for cardiovascular therapy (Romero and Duarte 2023; Gan et al. 2024; Hu et al. 2019). Therefore, gut microbiota has an impact on cardiovascular physiology, either directly or indirectly. This review explores the composition of gut microbiota and its changes in CVDs, the mechanistic roles of microbial‐derived metabolites in cardiovascular pathophysiology, and the pathways connecting gut dysbiosis to endothelial, cardiac fibroblast and cardiomyocyte dysfunction, inflammation, and immune responses. We also discuss therapeutic interventions targeting the gut‐heart axis, including dietary strategies, probiotics, and emerging microbiota‐based approaches. By delving into these complex interactions, we aim to offer a comprehensive understanding of how gut microbiota influences CVDs pathophysiology and highlights potential pathways for developing novel therapeutics in precision medicine.

## 2 Gut Microbiota Composition and Diversity in Cardiovascular Health

The composition and diversity of gut microbiota are critical determinants of cardiovascular health. Healthy gut microbiota is characterized by a balanced community of microorganisms, including beneficial bacteria (e.g., *Lactobacillus, Bifidobacterium*) and other commensals that support metabolic, immune, and vascular homeostasis (Zhu et al. 2020; Flori et al. 2024). Disruption of this balance can impair metabolic and immune functions, increasing susceptibility to disease (Yoo et al. 2020).

Cardiovascular factors, including aging, obesity, dietary patterns, and a sedentary lifestyle, have been shown to induce gut dysbiosis. Dysbiosis is associated with intestinal inflammation and reduced integrity of the gut barrier, which in turn increases circulating levels of bacterial structural components such as LPS and microbial metabolites TMAO that may facilitate the development of CVD (Novakovic et al. 2020). Another important CVD risk factor, cigarette smoke, can directly and indirectly alter the gastrointestinal barrier and upregulate enzymes involved in oxidative stress (Caliri et al. 2021; Gallucci et al. 2020). A recent study demonstrated that the gut microbiota composition differs between smokers and nonsmokers, with smokers having a higher relative abundance of Actinobacteria and Cyanobacteria than nonsmokers, and these changes may have an impact on cardiovascular risk (Sublette et al. 2020).

### 2.1 Healthy or Optimal Gut Microbiota Composition

A healthy host–microorganism balance must be respected to optimally perform metabolic and immune functions and prevent disease development; it is different for everyone. Optimal gut microbiota is typically dominated by Firmicutes, Bacteroidetes, Actinobacteria, Proteobacteria, Fusobacteria, and Verrucomicrobia (Rinninella et al. 2019). Also, the most abundant classes are Clostridia, Bacteroidia, Bifidobacteriales, Enterobacterales, and Lactobacillales. The most abundant families include *Bacteroidaceae, Lachnospiraceae, Ruminococcaceae, Odoribacteraceae, Rikenellaceae, Bifidobacteriaceae, Enterobacteriaceae*, and *Tannerellaceae*, etc. (Dixit et al. 2021).

### 2.2 Common Factors Influencing Gut Microbiota Composition

**FIGURE 1:**
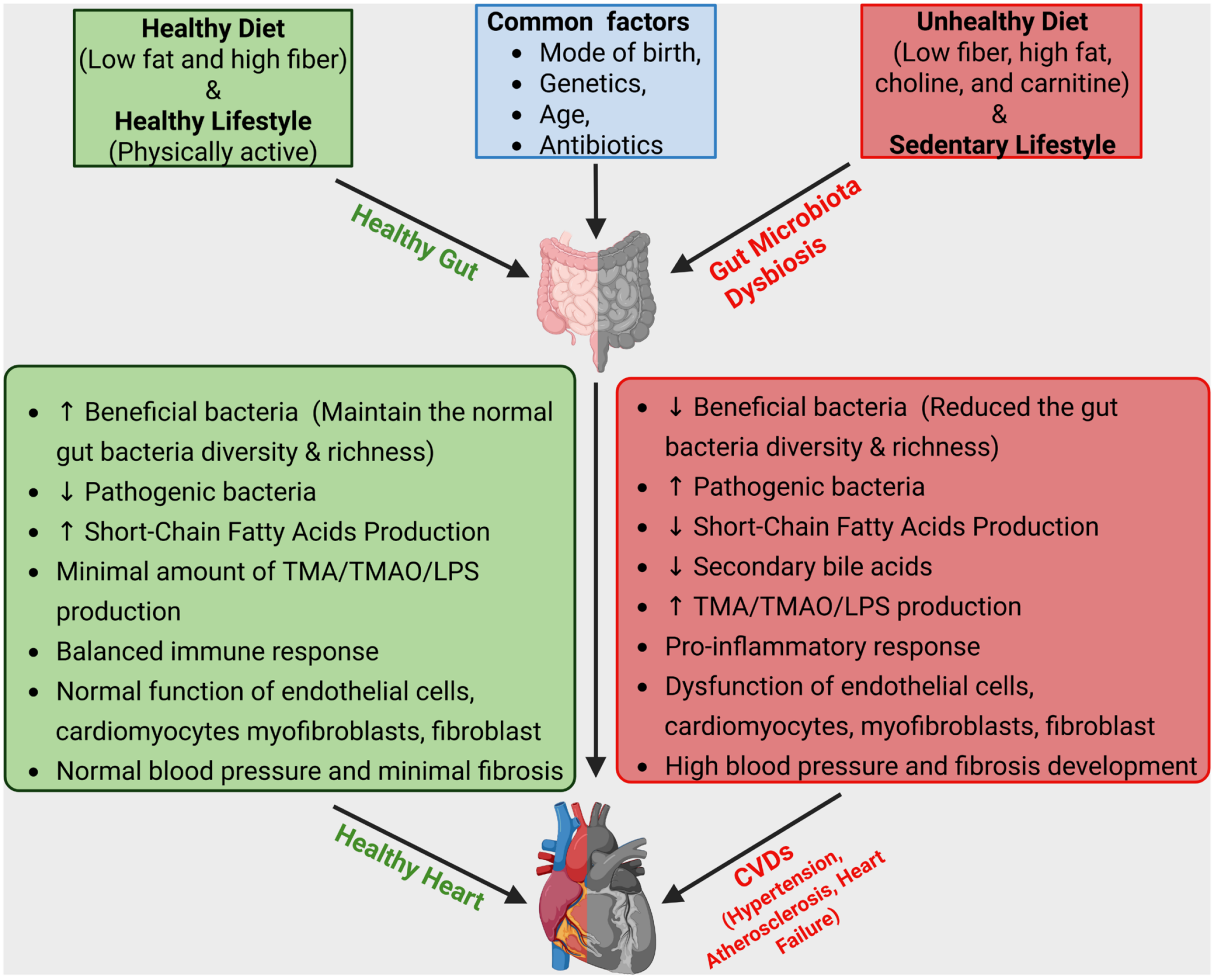
Impact of diet and lifestyle on gut microbiota and heart health. A healthy diet (low‐fat, high fiber) and an active lifestyle promote the beneficial gut bacteria growth, increase the helpful gut microbiota metabolites like SCFAs, and reduce the harmful gut microbiota metabolites like TMA/TMAO/LPS, maintain the normal blood pressure and heart function. An unhealthy diet (low fiber, high fat, high choline, and carnitine) and a sedentary lifestyle disrupt gut microbiota balance, increasing the harmful bacteria and gut microbiota metabolites while reducing the beneficial bacteria and gut microbiota metabolites. This leads to inflammation, high blood pressure, endothelial dysfunction, and cardiovascular diseases (CVDs). Other common factors like genetics, age, birth mode, and antibiotic use also affect gut bacteria and heart health. LPS, lipopolysaccharide; SCFAs, short‐chain fatty acids; TMA, trimethylamine; TMAO, trimethylamine‐N‐oxide.

Everyone possesses a unique gut microbiota influenced by various factors, particularly during early life (4–36 months). This period is critical for establishing core native microbiota, shaped by gut maturation, the development of enterotypes, birth gestational age, mode of delivery, feeding practices (e.g., breastfeeding or formula feeding), weaning, lifestyle, and dietary and cultural habits (Arrieta et al. 2015; Gensollen et al. 2016). By the age of 2–3 years, the gut microbiota composition typically achieves relative stability (Rodriguez et al. 2015). For example, age‐related shifts in gut microbiota have been well‐documented, with microbial diversity decreasing in the elderly (Odamaki et al. 2016; Jandhyala et al. 2015). Similarly, the mode of delivery at birth, such as vaginal delivery versus cesarean section, significantly impacts initial microbial colonization (Nagpal et al. 2017; Wen and Duffy 2017). The composition of the gut microbiota is significantly influenced by genetic and environmental factors, which have direct implications for CVD (Abdulrahim et al. 2025; Stock 2013). Interpersonal microbiome variability has a total of 22%–36% of its origins from environmental factors, and only 1.9%–19% is linked to genetics (Rothschild et al. 2018). Studies have shown a strong link between genetic loci and changes in gut microbiota (Kurilshikov et al. 2021). A genome‐wide analysis revealed that the long‐chain triglyceride locus encoding the enzyme lactase influenced the abundance of Bifidobacterium and this study suggests that dairy intake can modulate gut microbiota genes (Qin et al. 2024). Another study has shown that genetic variants that are linked to changes in gut microbiota are also connected to a higher chance of developing coronary artery disease (Khera et al. 2018). The gut microbiome is directly influenced by environmental factors such as diet, lifestyle, and antibiotics/drugs. The nature and abundance of microbial metabolites can have either positive or negative effects on cardiovascular health. Studies show that the microbiota composition of vegetarian diets is beneficial because they increase the Prevotella enterotype, while diets with high animal protein foster the Bacteroides enterotype and other species related to proatherogenic metabolites and CVD (David et al. 2014; Kahleova et al. 2020). Certain gut microbes can reduce the effectiveness of cardiovascular drugs like digoxin; it may not be efficacious when *Eggerthella lenta* strains are abundant, since they inactivate this drug, while some medications like metformin can, in turn, alter the gut microbiota by increasing harmful bacterial species like Escherichia‐Shigella (Zhao and Wang 2020). It has been reported that antibiotic‐induced gut dysbiosis can activate different pathways and potentially increase the risk of CVDs (Kaur et al. 2025). Antibiotic exposure is another critical factor, often disrupting microbial balance and reducing protective species (Goodrich et al. 2014; Ley et al. 2005; Turnbaugh et al. 2009). Antibiotics such as Azithromycin, Amoxicillin, Clindamycin, Vancomycin, Cephalexin, and Ciprofloxacin are commonly used to treat bacterial infections but *also affect the beneficial bacteria* and disrupt gut homeostasis by reducing diverse beneficial bacterial strains such as Bifidobacterium and Lactobacillus (Duan et al. 2022; Yang et al. 2021). Tetracycline is a bacteriostatic antibiotic which suppresses bacterial growth, but it does not kill them, resulting in excess proliferation of gram‐negative bacteria *E. coli* and overexpression of LPS‐related genes and reducing the diversity of Bifidobacterium (Breijyeh et al. 2020; Elvers et al. 2020). The use of broad‐spectrum antibiotics Penicillin and Amoxicillin can reduce the Bacteroides, Bifidobacterium, specifically Bifidobacterium adolescentis and *Bifidobacterium bifidum* , and Lachnospiraceae and Ruminococcaceae (Andrei et al. 2025). Clarithromycin reduces the population of Enterobacteriaceae, Bifidobacterium, and Lactobacillus, while macrolides increase the population of Proteobacteriae (Elvers et al. 2020; Korpela et al. 2016). Studies reported that the use of broad‐spectrum antibiotics can disrupt the balance of gut microbiota and make it more danger to the development of resistant species or pathobionts, such as *E. coli* , *Enterococcus faecalis* , and *Clostridium difficile* (Ianiro et al. 2016). The antibiotic usage reducing the SCFAs producing bacteria such as *Faecalibacterium prausnitzii* , and Roseburia destroys the mucus layer and disrupts tight junctions of the intestinal barrier, making the gut prone to infections (Zhang, Cheng, et al. 2021). Recent reports show that antibiotics‐induced gut dysbiosis can activate various pathways, potentially increasing the risk of CVDs through decreases in SCFAs, bile acid metabolism and increases in TMAO production, intestinal permeability allowing LPS and TMAO into systemic circulation (Kaur et al. 2025). Perturbations in the gut microbiota have been linked to various human diseases, including CVDs (Jie et al. 2017; Cui et al. 2017; Troseid 2017) and hypertension (Li et al. 2017), and other diseases such as IBD, obesity, diabetes, allergies, and autoimmune disorders (Hasan and Yang 2019). These associations highlight the critical role of gut microbiota in health and disease (Figure 1).

### 2.3 Gut Dysbiosis and CVD

Dysbiosis alters microbial diversity, diminishes beneficial taxa, and promotes pathogenic microorganisms, which can lead to systemic effects on metabolic, immune, and cardiovascular health (Table 1). Microbial signatures in dysbiosis include reduced abundance of SCFA‐producing bacteria (e.g., *Faecalibacterium prausnitzii* ) and beneficial genera like *Akkermansia* and *Roseburia* (Louis and Flint 2009; Geerlings et al. 2018), increased levels of opportunistic pathogens (*Enterobacteriaceae*, *Escherichia coli* ) and TMAO‐producing bacteria (*Lachnoclostridium*, *Anaerotruncus*) which are linked to cardiovascular risk (Tang and Hazen 2017; Romano et al. 2015). Understanding the factors that shape microbiota and the mechanisms linking dysbiosis to cardiovascular pathology offers new opportunities for targeted interventions to promote heart health.

**TABLE 1:** Gut dysbiosis and cardiovascular disease studies.

CVD condition	Microbiome changes	Metabolites changes	Ref
Atherosclerosis	↑ *Streptococcus anginosus* , *Atopobium parvulum* , *Actinomyces graevenitzii* , *Streptococcus mitis* /oralis/pneumonia. ↓ *Bacteroides xylanisolvens* , *Odoribacter splanchnicus* , *Eubacterium eligens* , *Roseburia inulinivorans* , *Roseburia intestinalis* .	↓Propionate, butyrate, and isobutyrate	(Yoo et al. 2022)
	↑ Streptococcus and Enterobacteriaceae	↓ SCFAs, ↑ TMAO	(Rodriguez et al. 2015)
Hypertension	↑ Acetobacteroides, Alistipes, Bacteroides, Barnesiella, Clostridium, Desulfovibrio, Megasphaera, Microvirgula, Parabacteroides. ↓ Lactobacillus, Olsenella, Paraprevotella, Prevotella, Romboutsia, Ruminococcus.	↓ SCFAs (butyrate), ↑ TMAO	(Mousa et al. 2022)
	↑ Catabacter, Veillonella, Clostridium, Oscillibacter, Robinsoniella. ↓ Akkermansia Ruminococcus Anaerovorax Sporobacter Asaccharobacter	↓ SCFAs	(Effendi et al. 2022)
	↑ Gut microbial richness, diversity, Lactobacillus and Clostridia.	↓ Vitamin B12	(Nesci et al. 2023)
	↑ Prevotella, Klebsiella ↓ microbial richness	↓ PSTN and HTN include phosphatidylserine (PS), 3,4,5‐trimethoxycinnamic acid, lysophosphatidylcholine (LysoPC), S‐carboxymethyl‐L‐cysteine, and lysophosphatidylethanolamine (LysoPE)	(Jandhyala et al. 2015)
	↑ Collinsella, Clostridiales, Clostridium. ↓ Bacteroides, Roseburia, Eubacterium, Faecalibacterium.	↓ Beta‐carotene	(Onyszkiewicz et al. 2019)
CVD	↑ Roseburia, Eubacterium, Faecalibacterium. ↓ Escherichia, Shigella, Bilophila, Hungatella.	↑ Sphingomyelin ↓ Glycochenodeoxycholate, cis‐aconitate, and uric acid	(Xu et al. 2022)
	↑ Firmicutes and Bacteroidetes, F/B ratio and Bacteroides.	↓ SCFAs	(Kimura et al. 2011)
CAD	↑ Escherichia‐Shigella, Enterococcus. ↓ Faecalibacterium, Subdoligranulum, Roseburia, *Eubacterium rectale* .	↑ TMAO, ↓ butyrate	(Ke et al. 2018)
	↓ *Faecalibacterium prausnitzii* and *Bacteroides fragilis* ↑ Enterobacteriaceae, Streptococcus, and Desulfovibrio	↑ plasma zonulin, TMAO, and IL‐1B ↓ IL‐10 and FOXP3 mRNA expression	(Bonaz et al. 2017)
	↑ Firmicutes phylum ↓Firmicutes phylum	↑ TMAO, Dietary phosphatidylcholine	(Gensollen et al. 2016)
	↑ Lactobacillales ↓Bacteroidetes	Not reported	(Gidron et al. 2007)
HFpEF	↓ Ruminococcus ↓Firmicutes/Bacteroides.	↓ SCFA	(Karbach et al. 2016)
HFrEF	↑ Streptococcus, Veillonella ↓SMB53	↓ SCFAs, Vit B5	(Mell et al. 2015)
Stable systolic HF	↑ Prevotella, Hungatella, Succinclasticum. ↓ Lachnospiracea, Blautia, Eubacteriumhalli, Ruminococcaceae, Faecalibacterium, Bifidobacteriaceae.	Not reported	(J. L. Pluznick 2017)
HF	↑ Actinobacteria, Bifidobacterium, Escherichia/Shigella. ↓ Megamonas	↑ TMAO ↓ Propionate and acetate	(Festi et al. 2014)
	↑ Candida, Campylobacter, Shigella, Yersinia.	↑ TNF‐α, INF‐γ ↓ IL‐10, Vit B12	(Wang, Yang, et al. )
Chronic HF	↓ Coriobacteriaceae, Erysipelotrichaceae, Ruminococcaceae, and Blautia.	↑ TMAO	(Aron‐Wisnewsky et al. 2021)
Acute HF	↓ *Eubacterium rectale* , *Dorea longicatena* Depletion of Faecalibacterium	↑ TMAO	(Baker et al. 2017)
Stable chronic HF	↑ *Ruminococcus gnavus* ↓ *Faecalibacterium prausnitzii*	↑TMAO	(Baker et al. 2017)

## 3 Microbial Metabolites and Cardiovascular Pathophysiology

**FIGURE 2:**
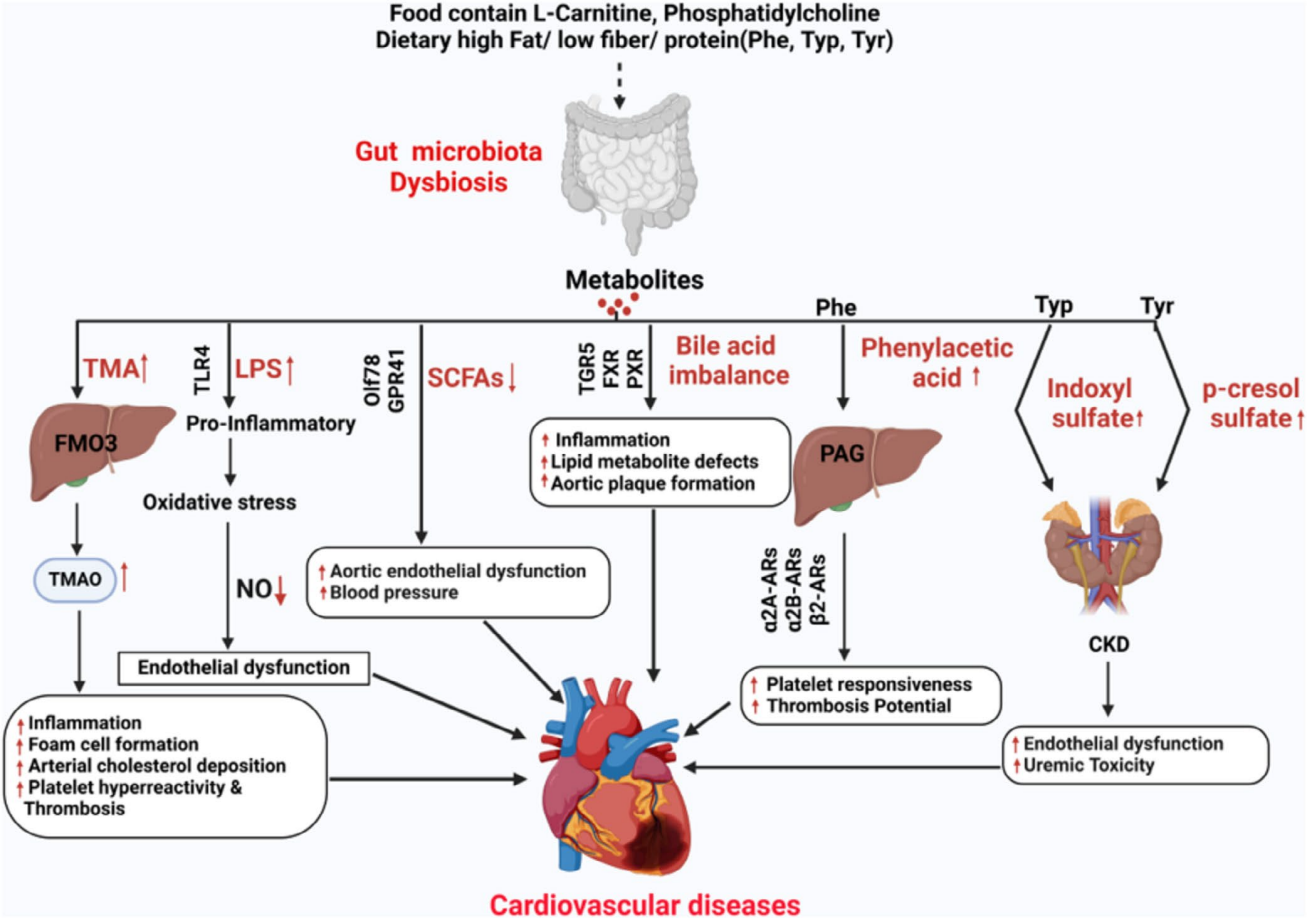
Microbial metabolites and their role in cardiovascular pathophysiology. High fat, low fiber foods and diet rich in phosphatidylcholine, choline, and L‐carnitine (such as red meat, cheese, seafood, egg yolk, etc.) is metabolized by specific gut microbiota to TMA which is further oxidized in the liver by FMOs to produce TMAO. TMAO contributes to CVD through several mechanisms such as inflammation, foam cell formation, arterial cholesterol deposition and platelet hyperreactivity and thrombosis. HFD induced gut dysbiosis can impair the gut barrier which leads to enhanced LPS or pathogens translocating into the circulation causing endotoxemia that stimulates the release of systemic pro‐inflammatory cytokines. These cytokines trigger damage of endothelial cells through interaction with TLR‐4 on cellular surface and enhance the generation of ROS to reduce endothelial NO bioavailability resulting in the formation of plaque and atherosclerosis lesion and thereby leads to CVD. A low‐fiber diet results in decreased production of SCFAs by gut bacteria. SCFAs help regulate blood pressure by activating receptors like Olf78 and GPR41. With fewer SCFAs, blood pressure rises, damaging the aortic endothelium and increasing the risk of CVD. Primary BAs are synthesized from dietary fats or cholesterol via enterohepatic circulation. BAs activate receptors like FXR and TGR5, which help regulate lipid metabolism, glucose levels, and inflammation. However, an imbalance in bile acids can disrupt these pathways, increasing the risk of CVD. Dietary proteins rich in aromatic amino acids such as Phe could be converted into phenylacetic acid via the gut microbiota and then transferred into PAG in the liver. PAG acts on G‐protein coupled receptors including α2A, α2B and β2‐ARs to facilitate platelet responsiveness, thrombosis potential to promote atherosclerotic leads to CVD. The other gut microbial derived metabolite: IS from Trp and pCS from Tyr could also predict CVD events in CKD patients which might be associated with uremic toxicity and endothelial dysfunction. Ars, adrenergic receptors; BAs, bile acids; CKD, chronic kidney disease; FMOs, flavin monooxygenases; FXR, Farnesoid X‐activated receptor; GPR41, G‐protein receptor 41; HFD, high fat diet; IS, indoxyl sulfate; NO, Nitric oxide; Olf78, olfactory receptor 78; PAG, phenylacetyl glutamine; pCS, p‐cresol sulfate; Phe, phenylalanine; PXR, Pregnane X receptor; ROS, reactive oxygen species; SCFAs, short‐chain fatty acids; TGR5, Takeda G protein‐coupled receptor 5; TLR‐4, Toll‐like receptor 4; TMA, trimethylamine; TMAO, trimethylamine‐N‐oxide; Trp, tryptophan; Tyr, tyrosine.

Trillions of microorganisms reside in the human gut, contributing not only to nutrient and energy extraction from ingested food but also to the production of bioactive metabolic signaling molecules that influence health and disease, including CVDs (Wang and Zhao 2018). Gut microbiota‐derived metabolites such as TMAO, SCFAs, bile acids (BAs), LPS, uremic toxins, phytoestrogens, and anthocyanins have been identified as key modulators of cardiovascular physiology and pathology (Wang and Zhao 2018) and more so, these have been implicated in the pathogenesis of CVD, when dysbiosis occurs in the gut (Figure 2).

Certain foods like red meat, cheese, seafood, and egg yolks contain phosphatidylcholine, choline, and L‐carnitine (Wang et al. 2011, 2019; Romano et al. 2015; Koeth et al. 2013). TMA lyase containing functional microbial CutC/D genes help convert choline into TMA (Witkowski et al. 2020; Gérard 2020), which then enters the bloodstream and is oxidized in the liver by flavin monooxygenases (mainly FMO3) to form TMAO. TMAO contributes to CVD, for instance atherosclerosis through several mechanisms such as inflammation, foam cell formation, arterial cholesterol deposition, platelet hyperreactivity and thrombosis. It increases pro‐inflammatory markers like IL‐6, cyclooxygenase 2 (COX‐2), TNF‐α, and IL‐1β while reducing IL‐10, an anti‐inflammatory cytokine (Yang, Li, et al. 2019; Seldin et al. 2016; Chen, Zheng, et al. 2017). It activates stress proteins (HSP70, HSP60), promoting scavenger receptor (SR‐A1, CD36) activation in macrophages, leading to oxidized LDL (ox‐LDL) uptake and foam cell formation (Wang et al. 2011; Koeth et al. 2013; Yang, Li, et al. 2019). TMAO suppresses RCT, causing cholesterol buildup in arteries and worsening plaques (Manco et al. 2010). TMAO could stimulate platelets to activate sub‐maximal stimulus including thrombin, adenosine diphosphate (ADP) and collagen as well as to induce the release of intracellular calcium resulting in platelet hyperresponsiveness (Zhu et al. 2016).

High fat diet induces gut dysbiosis and impairs the gut barrier, which enhances LPS or pathogen translocation into the circulation, causing endotoxemia that stimulates the release of systemic pro‐inflammatory cytokines (Cani et al. 2008; Manco et al. 2010). Once translocated in the bloodstream, endotoxin can trigger the damage of endothelial cells through interaction with Toll‐like receptor 4 (TLR‐4) on the cellular surface and enhance the generation of reactive oxygen species (ROS) to reduce endothelial nitric oxide (NO) bioavailability, resulting in the formation of plaque and atherosclerosis lesion (Manco et al. 2010; Incalza et al. 2018).

Short‐chain fatty acids like acetate, butyrate, and propionate are produced by gut bacteria when they ferment dietary fiber (Canfora et al. 2015) (Figure 2). SCFAs act through G‐protein‐coupled receptors (Olfr78 and GPR41) to regulate BP and blood vessel function (J. Pluznick 2014). Studies show that acetate supplementation improved gut health and reduced BP, cardiac fibrosis, and cardiac hypertrophy (Marques et al. 2017). Similarly, propionate protected against hypertension‐related heart damage, while butyrate‐producing bacteria reduced atherosclerosis (Bartolomaeus et al. 2019; Kasahara et al. 2018). Propionate lowered BP in mice, while acetate and butyrate improved blood vessel health by increasing NO availability (Robles‐Vera et al. 2020). A low‐fiber diet results in low production of SCFAs by gut bacteria. With fewer SCFAs, BP rises, damaging the aortic endothelium and increasing the risk of CVD. The core mechanisms of SCFA action in the gut–heart axis involve activation of G‐protein‐coupled receptor (GPCR), inhibition of histone deacetylase, and restoration of mitochondrial function (Yukino‐Iwashita et al. 2022). These pathways work together to regulate cardiac function and maintain cardiovascular health. Recent studies have found that SCFAs can provide cardio protection through different mechanisms including modulating BP, encouraging postinfarction cardiac repair, anti‐inflammation, and maintaining the gut barrier. SCFA modulating BP, for example, propionate induces renin secretion and thus elevates BP through binding to the Olfr78 receptor, and propionate also acts as a powerful hypotensive by binding to Gpr41 SCFA receptor, which is expressed in smooth muscle cells of small vessels (Pluznick et al. 2013). Promote postinfarction cardiac repair via inducing infiltration of CX3CR1+ monocytes in the peri‐infarct zone (Tang, Chen, et al. 2019). SCFA act as anti‐inflammation agents. For example, butyrate can reduce inflammation by inducing Foxp3+ Treg cell proliferation and suppressing Th17 cell generation by activating GPCR 43 (Sivaprakasam et al. 2016). SCFAs are also able to inhibit cardiac fibrosis via their anti‐inflammatory properties, which is a crucial pathological process in the development of HF (Palm et al. 2022). SCFAs play as a gut barrier‐protective role via activating the hypoxia‐inducible factor, butyrate to maintain the physiologic relative hypoxia state in colon epithelial mucosa, which is necessary in maintaining gut barrier function (Kelly et al. 2015). SCFAs serve as energy sources while inhibiting histone deacetylase‐regulated gene expression and activating GPCR signaling, leading to a boost in cardiac function (Wang, Li, et al. 2023). In vivo studies show that dietary supplementation of butyrate can prevent high‐fat diet‐induced obesity and insulin resistance and nonalcoholic fatty liver disease via mechanisms related to the promotion of energy expenditure and induction of mitochondrial function (Henagan et al. 2015; Gao et al. 2009; Vinolo et al. 2012; Mattace Raso et al. 2013). Butyric acid ameliorates rat myocardial fibrosis by regulating M1/M2 polarization of macrophages and promoting recovery of mitochondrial function in a transverse aortic constriction (TAC) model (Li et al. 2022).

Free fatty acid receptors (FFAR) are members of G protein‐coupled receptors such as FFA1 (GPR40), FFA2 (GPR 43), FFA3 (GPR41) and FFA4 (GPR120), ligands are FFAs with different carbon chain lengths (Bartoszek et al. 2020; Offermanns 2014). FFA receptor types are essentially the SCFA receptors (SCFARs), responding to all SCFAs, including acetic acid, propionic acid, and butyric acid. All FFA receptors are G protein‐coupled receptors (GPCRs) that play important roles in the regulation of metabolism, immunity, inflammation, hormone/neurotransmitter secretion and as well play role in physiology cardiovascular system (Lymperopoulos et al. 2022). FFAR1 is present in the human heart and is elevated in HF, but it is not observed in rodent hearts, it is critical for understanding the role of FFA signaling in the context of CVD. FFAR4 is expressed in tissues that are closely linked to cardiometabolic disease, including the rodent and human heart. It can also be found in cardiac myocytes, fibroblasts, and macrophages. FFA receptor 4 is a nutrient sensor for long‐chain fatty acids, like ω3‐polyunsaturated fatty acids, that attenuates metabolic disease and resolves inflammation. The recent study reported that the mechanism of the cardioprotective effects of FFAR4 in cardiac myocytes via FFAR4‐cPLA2a (cytosolic phospholipase A2a)‐18‐HEPE (18‐hydroxyeicosapentaenoic acid) signaling (Murphy et al. 2022). FFARs are also involved in the regulation of arterial functions, including the proliferation, differentiation, migration, apoptosis, inflammation, and angiogenesis of vascular endothelial cells (VECs) and vascular smooth muscle cells (VSMCs). A recent study shows that FFARs, primarily activated by long‐chain FA (palmitate, oleate, linoleate) and SCFA (acetate, butyrate, propionate), trigger intracellular signaling via G proteins and β‐arrestins in endothelial cells, thereby regulating arterial functions such as endothelial dysfunction, inflammation, angiogenesis, atherosclerosis, and BP (Yu et al. 2024).

Bile acids help digest and absorb dietary fats (Gerard 2013; Heaton 1969) (Figure 2). In the liver, primary BAs—cholic acid (CA) and chenodeoxycholic acid (CDCA)—are made from cholesterol with the help of enzymes like cholesterol 7a‐hydroxylase (CYP7A1), sterol‐27‐hydroxylase CYP27A1 and oxysterol 7a‐hydroxylase (CYP7B1), which are regulated by gut bacteria (Sayin et al. 2013). Most primary BAs conjugated to glycine or taurine and are reabsorbed and recycled, while the rest are modified by gut microbes (Dawson et al. 2009). Bacteria such as *Bifidobacterium and Lactobacillus* deconjugate BAs, while *Clostridium and Eubacterium* convert them into secondary BAs like lithocholic acid (LCA) and deoxycholic acid (DCA) (Yao et al. 2018; Ridlon et al. 2006) and other bacteria such as *Actinobacteria, Proteobacteria, Clostridium* further modify BAs through oxidation and epimerization (Kisiela et al. 2012; Lepercq et al. 2004). Once the microbial metabolized BAs enter the bloodstream, they activate receptors that regulate metabolism and influence CVDs. The nuclear receptor‐Farnesoid X Receptor (FXR) helps control lipid and glucose metabolism and protects against atherosclerosis by reducing plaque formation (Wahlstrom et al. 2016; Hartman et al. 2009). Takeda G protein‐coupled receptor 5 (TGR5) is activated by secondary BAs and reduces vascular inflammation, lowering plaque buildup (Pols et al. 2011). Pregnane X Receptor (PXR), activated by secondary BAs like LCA, also plays a role in BA metabolism (Zhou et al. 2009). In addition to FXR, TGR5, and PXR receptors, other receptors like liver X receptor (LXR) and vitamin D receptor (VDR), as well as G protein‐coupled receptors (muscarinic receptor, S1PR) and calcium‐activated potassium (K^+^) channels (BK), are present in heart and blood vessel cells. These include heart muscle cells, endothelial cells, cardiac fibroblasts, and VSMCs. When BAs bind to these receptors, they trigger changes inside the cells that can impact heart disease risk. Through these interactions, BAs play a role in the development of heart‐related conditions like cardiomyopathy, atherosclerosis, irregular heartbeats (arrhythmia), and HF by influencing key factors involved in cardiovascular health (Zhang et al. 2023). While most studies are based on mice, human studies showed that lower BA levels are linked to worse survival in HF patients and more severe coronary artery disease and heart attacks (Mayerhofer et al. 2017; Li, Shu, et al. 2020).

Aromatic amino acids (AAAs) like phenylalanine (Phe), tryptophan (Trp), and tyrosine (Tyr) are found in protein‐rich foods such as beef, pork, chicken, and fish (Zhang and Gerard 2022), (Figure 2). Recent studies have linked phenylacetyl glutamine (PAGln), a gut microbial metabolite from Phe, to major heart conditions like heart attacks (MI), strokes, and coronary artery disease (Nemet et al. 2020; Yu et al. 2021; Ottosson et al. 2020). Gut bacteria convert Phe into phenylacetic acid, which is then processed in the liver to form PAGln. PAGln activates GPCR (α2A, α2B, β2‐adrenergic receptors), increasing platelet activity and thrombosis risk (Nemet et al. 2020). PAGln circulating levels were elevated in aged humans and mice (Saeedi Saravi et al. 2025). A recent report in human patients demonstrated PAGln levels were significantly higher in the coronary heart disease (CHD) group compared to the control groups (You and Gao 2025). Mechanistically, PAGln can enhance platelet adhesion and thrombus formation via activation of adrenergic receptors and impose stable plaque formation leading to development of thrombosis and cardiovascular risk (You and Gao 2025). Similarly, gut microbes break down Trp into indoxyl sulfate (IS) and Tyr into p‐cresol sulfate (PCS) (Lin et al. 2012; Glorieux et al. 2021). These metabolites are linked to CVD, especially in chronic kidney disease (CKD) patients, by causing uremic toxic effects and blood vessel damage (Figure 2). Collectively, the gut microbiota‐derived metabolites play a crucial role in cardiovascular pathophysiology by regulating inflammation, lipid metabolism, and BP.

Other metabolites derived from gut microbial metabolism include indole derivatives, imidazole propionate (ImP), and succinate, which have been linked to risks of adverse cardiovascular events. Indole derivatives are microbial metabolites of Trp. Similar to PAGln, higher circulating levels of indole derivatives are associated with major adverse cardiovascular events. However, some studies also suggest that specific indole metabolites, such as indole‐3‐acetic acid (IAA) and indole‐3‐propionic acid (IPA), may have beneficial associations with cardiometabolic risk markers, including reductions in LDL cholesterol and improvements in vascular function (Mu et al. 2025). This highlights a complex, context‐dependent relationship between different indole derivatives and cardiovascular health. ImP is a gut microbiota‐derived metabolite from histidine metabolism. Elevated plasma ImP levels are consistently found in individuals with established CVD and HF and are associated with increased risk of onset of T2D and obesity, CKD, progression of atherosclerotic plaques, and elevated mortality rates in HF (Zeng et al. 2025). Further, ImP has also been shown to impair glucose metabolism and insulin signaling, beyond contributing to cardiometabolic risk (Zeng et al. 2025). Succinate is produced both by the host and gut microbiota. Elevated circulating succinate levels are observed in obesity, hypertension, ischemic heart disease, and T2D, all of which are major risk factors for CVD in young adults (Osuna‐Prieto et al. 2021). Mechanistically, succinate can promote oxidative stress in macrophages, exacerbate endothelial dysfunction, and activate the GPR91 (SUCNR1) receptor, which can stimulate renin release and contribute to fibrogenesis (Shan et al. 2022). These pathways link succinate to the development and progression of atherosclerosis and other cardiovascular complications.

## 4 Pathways Linking Gut Dysbiosis to CVDs

### 4.1 Systemic Inflammation and Dysfunction of Endothelial Cells, Cardiac Fibroblasts, and Cardiomyocytes

**FIGURE 3:**
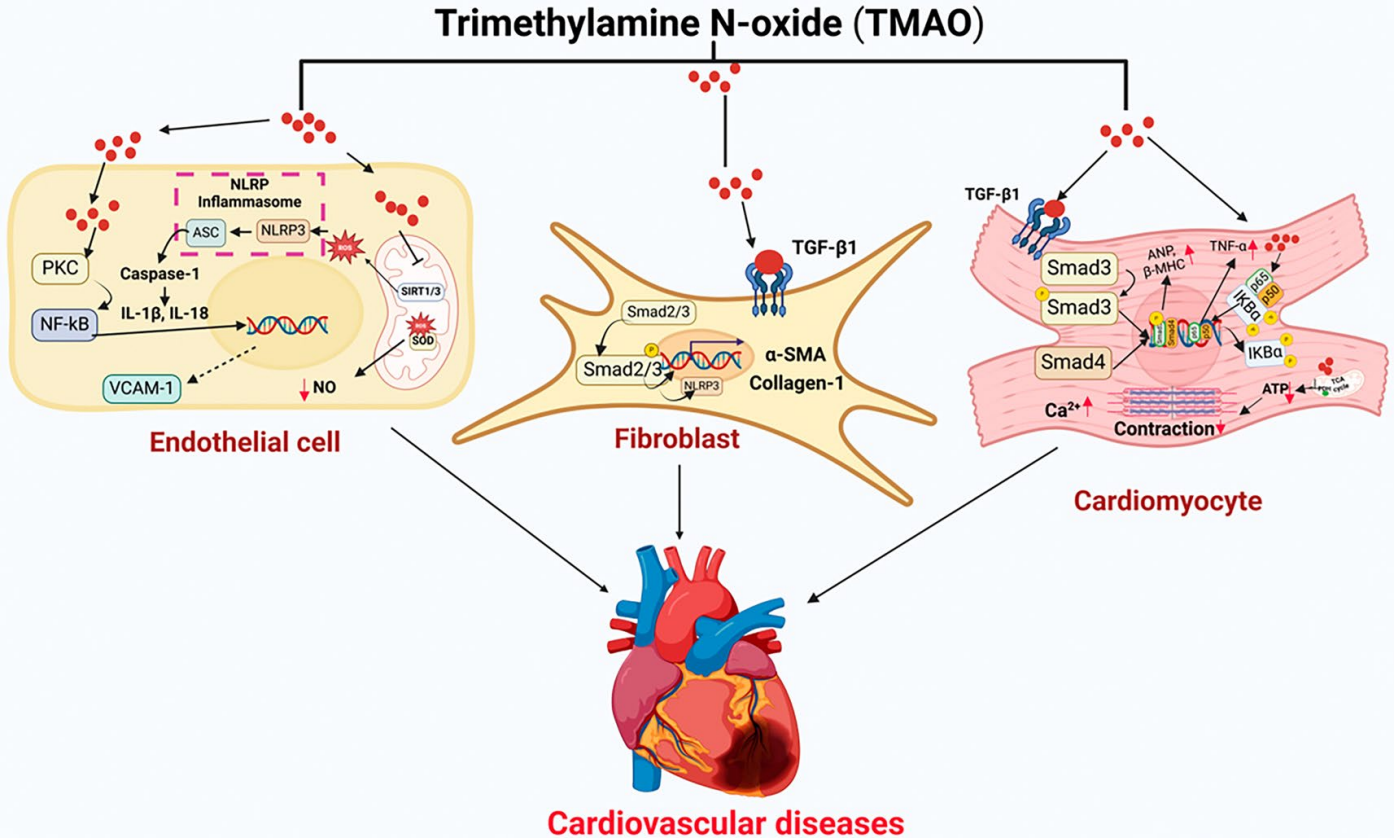
Pathways linking gut‐derived metabolite TMAO to CVDs via endothelial, cardiac fibroblast and cardiomyocytes dysfunction. TMAO in endothelium inhibits the expression SIRT3 and the activity of SOD2, leading to the accumulation of mtROS. This oxidative stress activates the NLRP3 inflammasome resulting in the production of pro‐inflammatory cytokines IL‐1β and IL‐18, which contribute to endothelial inflammation. Elevated circulating TMAO levels have been associated with decreased expression of SIRT1, leading to increased oxidative stress, resultant excessive ROS accumulation and reduced SIRT1 activity TMAO upregulates VCAM‐1 expression by activating the protein kinase C (PKC)/NF‐κB signaling pathway. This process directly results in endothelial dysfunction, characterized by reduced self‐repair capacity and increased monocyte adhesion to the endothelium. TMAO in cardiac fibroblasts activating the TGF‐βRI/Smad2/NLRP3 pathway and upregulation of α‐SMA and collagen I expression which leads to the conversion to cardiac myofibroblasts. TMAO in cardiomyocytes triggers TGF‐β1/Smad3 and p65 NF‐κB signaling pathways and lowers energy metabolism and mitochondrial function by disrupting pyruvate and fatty oxidation, as well as the TCA cycle. It also adversely affects myocardial contractile function and intercellular calcium processing. TMAO also increased the expression levels of ANP and β‐MHC, which caused myocardial hypertrophy and fibrosis through activation of the TGF‐β1/Smad3 pathway. ANP, atrial natriuretic factor; IL‐18, interleukin‐18; mtROS, mitochondrial ROS; NLRP3, nucleotide‐binding oligomerization domain‐like receptor family pyrin domain‐containing 3; SIRT1, sirtuin 1; SIRT3, sirtuin 3; SOD2, superoxide dismutase 2; β‐MHC, major histocompatibility complex.

One of the earliest consequences of systemic inflammation mediated by gut dysbiosis is endothelial dysfunction. Pro‐inflammatory cytokines and oxidative stress disrupt eNOS activity, reducing NO bioavailability. NO is essential for vascular homeostasis, mediating vasodilation, inhibiting leukocyte adhesion, and reducing platelet aggregation. Reduced NO levels lead to increased vascular stiffness and impaired blood flow (Maiuolo et al. 2022). Furthermore, inflammatory mediators upregulate adhesion molecules such as intercellular adhesion molecule‐1 (ICAM‐1) and vascular cell adhesion molecule‐1 (VCAM‐1). These molecules promote leukocyte adhesion and transmigration into the endothelium, exacerbating vascular inflammation and promoting atherosclerotic plaque development (Alexandrescu et al. 2024). Importantly, TMAO has been implicated in promoting endothelial inflammation and dysfunction through several mechanisms (Figure 3). TMAO inhibits the expression of sirtuin 3 (SIRT3) and the activity of superoxide dismutase 2 (SOD2), leading to the accumulation of mitochondrial ROS (mtROS). This oxidative stress activates the nucleotide‐binding oligomerization domain‐like receptor family pyrin domain‐containing 3 (NLRP3) inflammasome, resulting in the production of pro‐inflammatory cytokines IL‐1β and interleukin‐18 (IL‐18), which contribute to endothelial cell inflammation (Sun et al. 2016; Chen, Zhu, et al. 2017). Elevated circulating TMAO levels have been associated with decreased expression of sirtuin 1 (SIRT1), leading to increased oxidative stress. The resultant excessive ROS accumulation and reduced SIRT1 activity further impair NO production, adding to endothelial dysfunction (Ke et al. 2018). TMAO upregulates VCAM‐1 expression by activating the protein kinase C (PKC)/NF‐κB signaling pathway. This process directly results in endothelial dysfunction, characterized by reduced self‐repair capacity and increased monocyte adhesion to the endothelium (Ma et al. 2017).

Cardiac fibroblasts, which play a central role in maintaining the structural integrity of the heart, are also significantly affected by gut microbiota and their metabolites. Metabolites like TMAO and LPS promote the activation of cardiac fibroblasts into myofibroblasts. This transition is associated with increased extracellular matrix (ECM) deposition, leading to fibrosis and impaired myocardial compliance (Costa et al. 2022). Chronic systemic inflammation driven by gut dysbiosis enhances the secretion of pro‐inflammatory cytokines, such as IL‐6 and TGF‐β, which stimulate fibroblast activation and collagen synthesis. This contributes to pathological remodeling and heart stiffness (Melendez et al. 2010; Lijnen et al. 2000; Annes et al. 2003). Numerous animal studies have suggested that elevated TMAO levels are strongly linked to cardiac fibrosis and contribute to HF (Chen, Zheng, et al. 2017; Zhang et al. 2017; Li, Wu, et al. 2019). The profibrotic mechanisms of TMAO in the myocardium closely resemble those observed in the kidney, primarily involving TGF‐β and the NLRP3 inflammasome (Li, Geng, et al. 2019). Treatment of primary mouse cardiac fibroblasts with TMAO resulted in a dose‐dependent increase in proliferation, migration, collagen secretion, and the expression of profibrotic factors, including TGF‐β and phosphorylated SMAD3 (Li, Geng, et al. 2019). Additionally, TMAO exposure enhanced NLRP3 inflammasome activation in cardiac fibroblasts, whereas siRNA‐mediated knockdown of NLRP3 attenuated TMAO‐induced proliferation, as well as TGF‐β and collagen expression (Li, Geng, et al. 2019). A recent study demonstrates that TMAO or a high choline diet aggravates cardiac function and the transformation of fibroblasts into myofibroblasts by activating the TGF‐βRI/Smad2 pathway. It enhanced TGF‐β receptor I expression, leading to increased Smad2 phosphorylation and upregulation of α‐SMA and collagen I expression (Yang, Zhang, et al. 2019) (Figure 3). Furthermore, TMAO has been shown to drive the transformation of atrial fibroblasts into myofibroblasts via activation of the Wnt2a/β‐catenin signaling pathway (Yang, Zhang, et al. 2019; Yang, Qu, et al. 2019).

Recent studies suggest that gut‐derived metabolites, TMAO, SCFAs, and secondary BAs significantly impact cardiomyocyte physiology and pathology through various mechanisms (Zhang, Wang, et al. 2021). Elevated TMAO levels are associated with adverse cardiovascular outcomes, including HF, arrhythmias, and myocardial fibrosis (Zhen et al. 2023). TMAO in cardiomyocytes triggers TGF‐β1/Smad3/p65 NF‐κB signaling pathways and lowers energy metabolism and mitochondrial function by disrupting pyruvate and fatty oxidation, as well as the TCA cycle. TMAO also increased the expression levels of atrial natriuretic factor (ANP) and β‐myosin heavy chain (β‐MHC), which caused myocardial hypertrophy and fibrosis through activation of the TGF‐β1/Smad3 pathway (Li, Wu, et al. 2019) (Figure 3). It also adversely affects myocardial contractile function and intercellular calcium processing (Li, Wu, et al. 2019; Makrecka‐Kuka et al. 2017; Savi et al. 2018). Recent studies highlight TMAO's complex effects on mitochondrial function and cardiomyocyte health (Wang et al. 2011; Tang et al. 2013). TMAO impairs mitochondrial function in cardiomyocytes by disrupting mitochondrial respiration and OXPHOS, leading to reduced ATP production and energy metabolism (Li et al. 2023). It also increases mitochondrial oxidative stress by inhibiting key antioxidant enzymes and promotes inflammation and induces cardiomyocyte apoptosis (Li et al. 2023). However, these effects are dose and time dependent, and more research is needed to explore specific mechanisms of TMAO.

On the other hand, as mentioned above, SCFAs, particularly butyrate and propionate, exhibit cardioprotective effects by enhancing mitochondrial efficiency, reducing ROS production, and attenuating inflammatory responses in cardiomyocytes. These actions preserve myocardial function and limit ischemic injury (Wang and Zhao 2018; Yukino‐Iwashita et al. 2022; Li et al. 2012; Kessler‐Icekson et al. 2012). These findings suggest that gut dysbiosis contributes to CVD through enhanced systemic inflammation inducing endothelial dysfunction, fibroblast activation, and cardiomyocyte impairment, eventually leading to CVD.

### 4.2 Immunomodulation

The gut microbiota plays a crucial role in shaping the immune system, influencing both local and systemic immune responses. Dysbiosis disrupts this balance, leading to heightened immune activation and chronic low‐grade inflammation—a recognized driver of CVD. In a healthy state, the gut microbiota promotes immune tolerance through Treg activation and the production of anti‐inflammatory cytokine IL‐10, thus maintaining immune homeostasis and preventing excessive inflammation. Dysbiosis reduces the abundance of beneficial microbes like *Faecalibacterium prausnitzii* and *Akkermansia muciniphila* , which are critical for maintaining immune homeostasis (Zheng et al. 2020; Yoo et al. 2022; Mousa et al. 2022; Effendi et al. 2022). This imbalance skews the immune response toward a pro‐inflammatory state, characterized by elevated levels of pro‐inflammatory cytokines such as IL‐6 and TNF‐α (Zheng et al. 2020). Gut dysbiosis increases the translocation of microbial components such as LPS, peptidoglycans, and bacterial DNA into systemic circulation due to impaired intestinal barrier function. These components activate pattern recognition receptors (PRRs), including TLRs and NLRs, on immune cells. As mentioned before, LPS binds to TLR4, triggering a cascade of pro‐inflammatory signaling pathways that amplify systemic inflammation and contribute to endothelial dysfunction (Yoo et al. 2020; Maiuolo et al. 2022; Zheng et al. 2020). In addition to innate immune activation, dysbiosis modulates adaptive immunity by altering antigen presentation and T‐cell polarization. Increased microbial translocation promotes the activation of effector T‐cells, particularly Th17 cells, which secrete interleukin‐17 (IL‐17), a cytokine implicated in vascular inflammation and atherosclerosis (Zheng et al. 2020; Nesci et al. 2023).

### 4.3 Crosstalk via Neurohumoral and Hormonal Pathways

The gut microbiota plays a crucial role in modulating cardiovascular health through its interactions with neurohumoral and hormonal pathways (Singh et al. 2024). Previous investigations have revealed SCFA‐mediated sympathetic nerve activity by activating GPR41, which in turn is linked to the regulation of BP and hypertension (Onyszkiewicz et al. 2019; Xu et al. 2022; Kimura et al. 2011). Additionally, the vagus nerve serves as a crucial communication pathway between the gut and the brain, playing a significant role in regulating cardiovascular function and anti‐inflammatory signaling. Gut dysbiosis reduces vagal tone, impairing its ability to regulate inflammatory responses. Decreased vagal activity results in unchecked inflammation, promoting vascular damage and atherosclerosis (Bonaz et al. 2017; Gidron et al. 2007). Gut dysbiosis has been linked to the dysregulation of the renin‐angiotensin‐aldosterone system (RAAS), a critical regulator of BP and fluid homeostasis. Studies suggest that altered gut microbiota composition influences angiotensin II levels, promoting vasoconstriction, oxidative stress, and inflammation—hallmarks of hypertension and CVD (Tang et al. 2017; Karbach et al. 2016; Mell et al. 2015). Additionally, gut microbiota significantly affects metabolic hormones such as insulin, glucagon‐like peptide‐1 (GLP‐1), and ghrelin. Alterations in GLP‐1 production impair glucose metabolism and lipid homeostasis, increasing the risk of metabolic syndrome (Festi et al. 2014). Furthermore, disruption in the secretion of ghrelin indirectly influences cardiovascular risk factors such as obesity and hypertension (Aron‐Wisnewsky et al. 2021). Gut dysbiosis affects estrogen metabolism through the gut‐liver axis. Microbial beta‐glucuronidase enzymes modulate circulating estrogen levels, and dysbiosis can lead to hormonal imbalances that influence vascular function and inflammation, particularly in postmenopausal women at increased risk for CVD (Baker et al. 2017). Thus, the interplay between gut dysbiosis and neurohumoral and hormonal pathways underscores its pivotal role in CVD pathogenesis.

## 5 Gut Microbiota and Specific Cardiovascular Conditions

Although the gut microbiota influences the spectrum of CVDs, it has emerged as a significant factor in specific CV conditions, including hypertension, HF, atherosclerosis, and inflammation.

### 5.1 Hypertension and Microbial Metabolites

Hypertension, defined as persistent high BP, is a significant global health challenge associated with an increased risk of CVDs (Mills et al. 2020). Emerging evidence suggests that the gut microbiota plays a crucial role in the regulation of hypertension pathogenesis through its metabolites, particularly SCFAs and TMAO and secondary BAs (Poll et al. 2020; Ishimwe et al. 2022). As mentioned previously, SCFAs, primarily butyrate, propionate, and acetate, are produced by the fermentation of dietary fiber by gut microbiota (Rios‐Covian et al. 2016). Butyrate, produced by bacteria such as *Clostridium*, *Eubacterium*, and *Roseburia*, has multiple mechanisms contributing to BP regulation. It activates GPR41 and GPR43 on VECs, thereby reducing vascular resistance and lowering BP (Gerard 2013; Heaton 1969); mitigates systemic inflammation and oxidative stress, both of which are central to the development of hypertension (Mayerhofer et al. 2017); maintains the gut barrier by preventing the translocation of pro‐inflammatory substances into the bloodstream, thereby reducing factors that elevate BP (Li, Shu, et al. 2020); and modulates serotonin release from enterochromaffin cells (ECCs), enhancing gut‐brain axis signaling and influencing CNS mechanisms involved in BP regulation (Zhang and Gerard 2022). Acetate produced by bacteria such as *Faecalibacterium* and *Roseburia*, plays a pivotal role by serveing as a precursor for butyrate production and also independently induces vasodilation, reducing vascular resistance (Marques et al. 2017; Cookson 2021); enhances serotonin release from EECs by modulating the gut‐brain axis to regulate BP (Cookson 2021). Acetate supplementation in the drinking water of deoxycorticosterone acetate (DOCA)‐salt hypertensive mice resulted in reduced systolic and diastolic BP, as well as attenuation of cardiac fibrosis and hypertrophy (Marques et al. 2017). Acetate further reduces mean arterial pressure (MAP) and heart rate (HR) by influencing the autonomic nervous system, including reducing sympathetic tone and cardiac contractility (Poll et al. 2020). Propionate, generated by bacteria like *Veillonellaceae* and *Prevotella*, provides additional cardiovascular benefits such as reverses T‐cell imbalances caused by hypertension, aiding in cardiac remodeling (Bartolomaeus et al. 2019), activates GPR41 and GPR43 on cardiac fibroblasts, inhibiting myofibroblast formation and reducing collagen production, thereby protecting against fibrosis and vascular dysfunction. Furthermore, acetate and propionate jointly reduce cardiac hypertrophy, vascular dysfunction, and immune cell infiltration, improving heart function under stress (Lin et al. 2022).

In contrast to SCFAs, TMAO has been implicated in hypertension and other cardiovascular disorders. As described in the above section, elevated levels of TMAO are strongly linked to adverse cardiovascular outcomes, including endothelial dysfunction, atherosclerosis, and thrombosis. TMAO plays a pivotal role in endothelial dysfunction by driving inflammation, increasing oxidative stress, and impairing vascular reactivity—key mechanisms that contribute to the development and progression of hypertension. Mechanistically, TMAO promotes oxidative stress and impairs NO signaling, leading to vasoconstriction and hypertension (Shanmugham et al. 2023).

### 5.2 Heart Failure and Gut Permeability

HF is a complex clinical syndrome characterized by the heart's inability to pump sufficient blood to meet the body's needs. Emerging evidence highlights that HF is associated with alterations in gut microbiota composition, favoring pro‐inflammatory and pathogenic species. The “gut hypothesis of heart failure” proposes that HF‐induced factors, including reduced intestinal perfusion and altered gut motility, disrupt gut microbiota composition, and increase intestinal permeability. These conditions further compromise tight junctions, weakening the gut barrier's integrity (Matacchione et al. 2024; Harikrishnan 2019; Sandek et al. 2008). Concurrently, venous congestion causes intestinal edema, exacerbating epithelial barrier dysfunction (Sandek et al. 2008). Oxidative stress, another hallmark of HF, damages epithelial cells and impairs the synthesis of protective mucins, thereby aggravating gut barrier breakdown (Yuzefpolskaya et al. 2020). This leads to a compromised intestinal barrier, a selective interface between the host and gut microbiota, resulting in increased gut permeability. As mentioned before, the alteration in gut permeability leads to an increased passage of pathogens and toxins, such as LPS, from the gut into the systemic circulation. This state of “leaky gut” is associated with chronic systemic inflammation, characterized by elevated pro‐inflammatory cytokines, including TNF‐α, IL‐6, and IL‐1β, which contribute to myocardial remodeling and cardiac dysfunction and can exacerbate HF symptoms and severity (Tang et al. 2017; Lupu et al. 2023; Pasini et al. 2016).

### 5.3 Atherosclerosis and Inflammation

Atherosclerosis is a chronic, progressive disease characterized by the accumulation of lipids, inflammatory cells, and fibrous elements in the arterial wall, leading to the formation of plaques. These plaques can block blood flow and lead to heart attacks, strokes, and other cardiovascular problems (Lusis 2000). In recent years, multiple studies have confirmed the presence of bacterial DNA in atherosclerotic plaques, suggesting a potential role in the development of CVD (Ott et al. 2006). Additionally, differences in gut microbiota composition have been observed between individuals with and without atherosclerosis. Garshick et al. transplanted aortas from atherosclerotic Apoe^−/−^ mice into normolipidemic wild‐type (WT) recipients, followed by antibiotic treatment. Although plaque size remained similar to that in Apoe^−/−^ donors, there was a 32% reduction in CD68^+^ macrophages, suggesting that antibiotics delay atherosclerosis inflammation resolution and that gut microbiota play a role in atherosclerosis inflammation (Garshick et al. 2021). Studies found that patients with CHD or elevated intima‐media thickness (IMT), a marker of subclinical atherosclerosis, exhibited a higher *Firmicutes/Bacteroidetes* ratio, which is often linked to obesity and gut dysbiosis—highlighting the protective role of butyrate in CVD (Cui et al. 2017; Szabo et al. 2021). Additionally, other studies reported an enrichment of Escherichia in individuals with subclinical carotid atherosclerosis (SCA) and coronary artery disease (CAD), suggesting its potential as a predictive marker for atherosclerosis progression (Zhu et al. 2018; Baragetti et al. 2021). Ji et al. further identified an increased abundance of *Acidaminococcus* in patients with carotid atherosclerosis (CAS), a genus linked to inflammatory diseases and proinflammatory diets, suggesting its possible role as a proinflammatory microbiota in atherosclerosis development (Ji et al. 2021). Mitra et al. reported significant differences in gut microbiota composition between patients with stable versus unstable plaques (Mitra et al. 2015), whereas Hallenius et al. found no major differences in bacterial DNA content or microbial composition between the two. Some researchers suggest that bacterial DNA may activate macrophages and trigger the innate immune system via TLR2 and TLR4, which influence plaque stability (Mitra et al. 2015; Lindskog Jonsson et al. 2017).

Further research by Chen et al. demonstrated the feasibility of gut microbiome remodeling to prevent the onset and progression of atherosclerosis in LDLr^−/−^ mice, reinforcing the potential role of gut microbiota in atherosclerosis and its therapeutic implications. Additionally, one study found that introducing proinflammatory Casp1^−/−^ microbiota into LDLr^−/−^ mice promoted plaque growth, neutrophil accumulation, and a reduction in SCFAs‐producing taxa (Akkermansia, Christensenellaceae, Clostridium, and Odoribacter) (Chen et al. 2020).

## 6 Therapeutic Approaches Targeting the Gut‐Heart Axis

**FIGURE 4:**
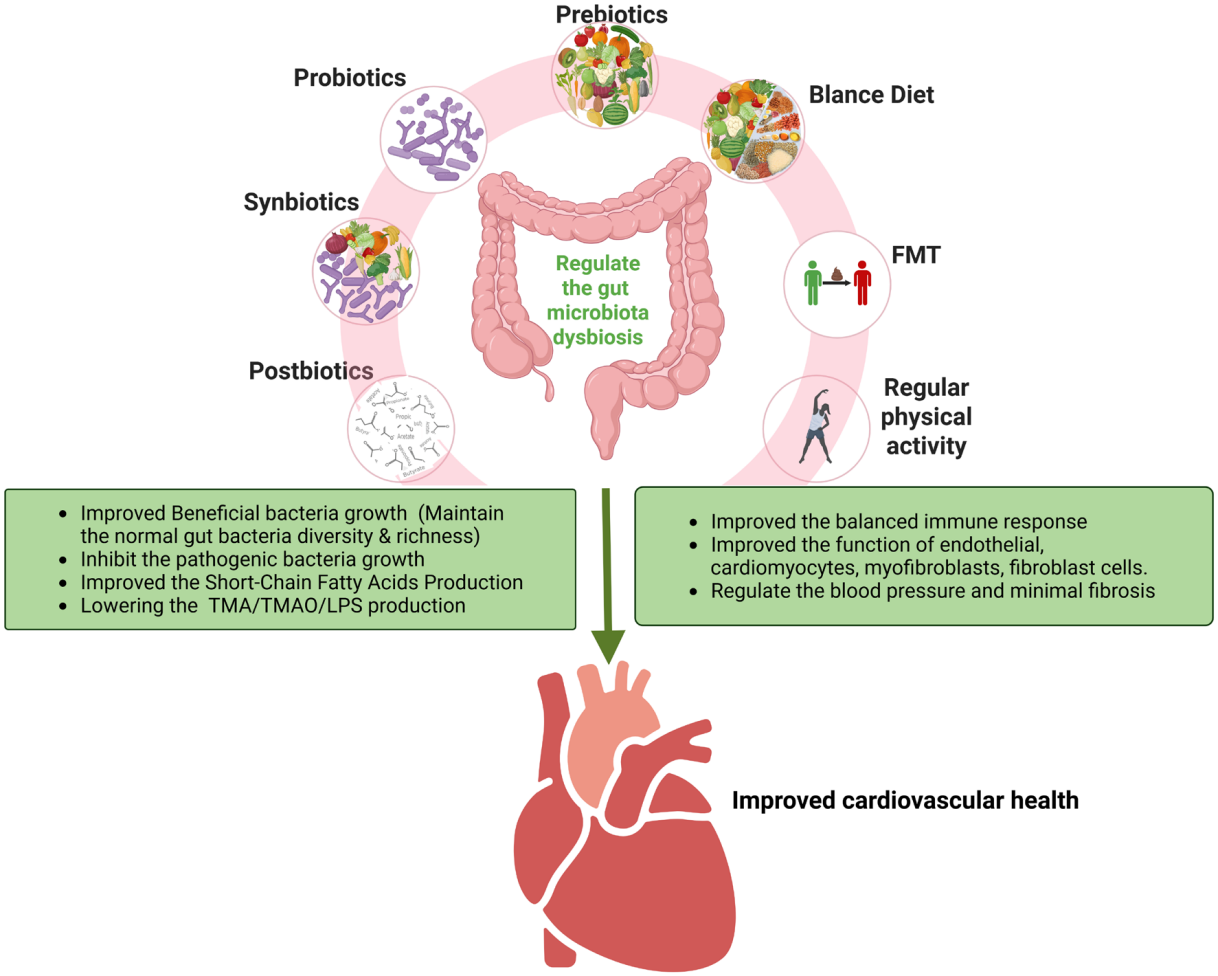
Therapeutic targeting of gut microbiota dysbiosis and its impact on cardiovascular health. Interventions such as prebiotics, probiotics, synbiotics, postbiotics, a balanced diet, regular physical activity, and fecal microbiota transplantation (FMT), help maintain gut microbiota homeostasis. These interventions promote beneficial bacterial growth, inhibit pathogenic bacteria, enhance the beneficial gut microbiota metabolites like SCFAs production, and reduce the harmful gut microbiota metabolites such as TMA, TMAO, and LPS. They also contribute to a balanced immune response, improved endothelial and cardiac cell function, regulated blood pressure, and reduced fibrosis, ultimately leading to improved cardiovascular health. SCFA‐short‐chain fatty acid; FMT, fecal microbiota transplantation.

Targeting the gut‐heart axis presents a promising therapeutic approach for mitigating the various CVD conditions. In this section of the review, we discuss various therapeutic strategies aimed at restoring gut homeostasis and their potential benefits for cardiovascular health (Figure 4).

### 6.1 Probiotics, Prebiotics, and Synbiotics

Probiotics are live microorganisms that confer health benefits to the host when administered in adequate amounts. Specific strains of *Lactobacillus* and *Bifidobacterium* have demonstrated the ability to reduce gut permeability, suppress inflammation, and improve lipid profiles—all critical factors in cardiovascular health. For instance, *Lactobacillus plantarum* supplementation reduced BP and improved endothelial function in preclinical models (Malik et al. 2018). Probiotics also lower TMAO levels by modulating microbial populations responsible for TMA production, such as *Lactobacillus plantarum ZDY04* (Qiu et al. 2018). Prebiotics, such as inulin and fructooligosaccharides, serve as substrates for beneficial gut bacteria. These compounds enhance the production of SCFAs like butyrate, which have anti‐inflammatory and vasodilatory effects. In vitro and in vivo studies suggest that the anti‐inflammatory and immunomodulatory properties of herbs and functional foods, such as *Ocimum sanctum, Zingiber officinale*, and *Piper nigrum* , may be linked to their prebiotic activity (Babu et al. 2018; Kondapalli et al. 2022). This is attributed to the presence of phytochemicals that help regulate gut microbiota, thereby reducing systemic inflammation and related disorders. Clinical studies show that prebiotic supplementation improves arterial stiffness and lowers systemic inflammation, thereby reducing cardiovascular risk (Pavlidou et al. 2022). While whole foods rich in natural fibers support beneficial gut bacteria and offer numerous health benefits, however, a recent study reveals that consumption of highly refined fermentable fibers may adversely affect liver health, leading to the development of icteric hepatocellular carcinoma (Singh et al. 2018). These discrepancies point to the fact that the activity of gut metabolites and signaling mediators is highly context‐dependent, influenced by variables such as dietary inputs, gut microbiota composition, host health status, and metabolic individuality. These factors collectively determine whether a metabolite exerts beneficial, neutral, or detrimental effects.

Synbiotics combine probiotics and prebiotics to synergistically enhance gut health. By delivering beneficial microbes and their specific growth substrates, synbiotics optimize the gut microbiota's functionality. Synbiotics leverage the complementary effects of probiotics and prebiotics, enhancing colonization resistance, SCFA production, and immunomodulation. Synbiotics have demonstrated that regular consumption of synbiotic yogurts can lower the risk of CVDs in hypercholesterolemic patients (Pavlidou et al. 2022). Additionally, a study found that 12 weeks of synbiotic supplementation resulted in a reduction of ICAM‐1 levels, a key risk factor for CVDs, in hemodialysis patients (Haghighat et al. 2019). All these interventions offer promising avenues for modulating gut microbiota, reducing inflammation, improving lipid profiles, and ultimately mitigating cardiovascular risk.

### 6.2 Dietary Interventions

The Mediterranean diet, rich in fiber, polyphenols, and unsaturated fats, promotes a healthy gut microbiome and reduces cardiovascular risk factors. Increased fiber intake supports the growth of SCFA‐producing bacteria, enhancing gut barrier integrity and reducing systemic inflammation. Dietary modifications, such as reducing red meat and egg consumption, can lower TMAO levels. This, in turn, mitigates endothelial dysfunction and atherosclerotic progression linked to TMAO (Abrignani et al. 2024).

### 6.3 Pharmacological Approaches

New pharmacological agents aim to selectively modulate gut microbiota composition. Drugs targeting TMAO production, such as 3,3‐dimethyl‐1‐butanol (DMB), inhibit microbial enzymes involved in TMA formation. DMB has shown efficacy in reducing TMAO levels and attenuating atherosclerosis in animal models (Wang et al. 2015). Direct supplementation of SCFAs, particularly butyrate, has been explored for its cardioprotective effects (Challa and Lewandowski 2022). Butyrate improves endothelial function, reduces oxidative stress, and modulates BP regulation via G protein‐coupled receptor activation (Amiri et al. 2021). Antibiotics targeting specific TMA‐producing bacteria have shown promise in modulating TMAO levels and potentially influencing cardiovascular outcomes. In healthy participants, administration of broad‐spectrum antibiotics significantly reduced plasma TMAO levels after a phosphatidylcholine challenge. However, it is crucial to note that chronic antibiotic use may have adverse consequences, such as the emergence of antibiotic‐resistant bacterial strains and potential metabolic disturbances (Yang, Li, et al. 2019). Bile acid modulators represent another class of drugs that can influence cardiovascular outcomes by altering gut microbiota composition and function. These agents can affect bile acid pool size and composition, which in turn impacts cholesterol metabolism and TMAO production. By modulating bile acid signaling through FXR, these drugs may offer a novel approach to managing CVD risk (Nesci et al. 2023).

Several established pharmacotherapies for CVDs are now recognized to alter the gut microbiome, with growing evidence that these interactions may influence both drug efficacy and cardiovascular outcomes. The captopril is a first‐generation angiotensin‐converting enzyme inhibitor (ACE‐Is); which not only lowers BP but also alters the gut microbiota composition. In a preclinical study, captopril‐induction, reduces the BP and alters the gut microbiome in spontaneously hypertensive (SHR) rats (Yang, Aquino, et al. 2019). Maternal captopril treatment led to persistent antihypertensive effects in offspring via reconstitution of the gut microbiota, including increased Clostridia and Clostridiales (Li, Yang, et al. 2020). Benazepril and Enalapril are the second‐generation ACE‐Is also impact the gut microbiome. Benazepril promotes restoration of gut microbiota structure, while enalapril has been shown to reduce blood levels of TMAO. Enalapril achieves this by modifying gut microbiota composition and increasing urinary excretion of methylamines (Zhao et al. 2023). Statins (e.g., simvastatin, rosuvastatin, atorvastatin) are widely used lipid‐lowering agents. Metagenomic studies have shown that statin use is associated with significant shifts in gut microbiota composition (Wilmanski et al. 2022). Statins may also decrease the amount of secondary BAs and alter signaling through the FXR, which is influenced by gut microbial metabolism (Tuteja and Ferguson 2019). Antihypertensive medications such as, amlodipine, a calcium channel blocker is metabolized presystemically by gut microbial dehydrogenation, which may reduce the amount of active drug reaching target tissues. Its use has also been associated with changes in gut microbiota, though the specific bacterial taxa involved are not fully characterized. Other cardiovascular drugs including, β‐blockers and α‐blockers have found associations between the use of these antihypertensive agents and alterations in gut bacterial taxa, though the precise mechanisms and clinical implications remain under investigation. An antidiabetic agent, metformin is frequently used in patients with CVD and has been shown to significantly alter the gut microbiota. It increases the abundance of beneficial bacteria such as *Akkermansia muciniphila* , which is linked to improved metabolic and cardiovascular outcomes (Witkowski et al. 2020; Masenga et al. 2022).

### 6.4 Fecal Microbiota Transplantation and Emerging Strategies

Fecal microbiota transplantation involves transferring processed stool from a healthy donor into the gastrointestinal tract of a patient, aiming to restore a balanced and diverse gut microbiome (Ponce Alencastro et al. 2025). This intervention is based on the principle that many diseases, especially those involving gut dysbiosis, can be improved by reintroducing beneficial microbial communities (Cammarota et al. 2014). The therapeutic effect of FMT is thought to arise from: (i) Replacing missing or depleting beneficial bacteria; (ii) Competing with pathogenic microbes (bacterial interference); (iii) Restoring production of key metabolites (e.g., SCFAs, secondary BAs); (iv) Modulating immune system and metabolic pathways. Clinically, FMT has shown some beneficial effects for the treatment of *Clostridium difficile* (CD)‐associated diarrhea (Cammarota et al. 2014). However, the studies that show the effects of FMT on metabolic parameters remain controversial. A meta‐analysis report on cardiometabolic risk factors demonstrated that no significant changes were seen in lipid profiles, blood glucose, and insulin resistance (Pakmehr et al. 2024). However, various other studies were reported with changes in the lipid profile including reduction in LDL (*p* < 0.04) and total cholesterol (*p* < 0.05). Mean triglycerides (TG) were reduced significantly from 3.9 mmol/L to 2.62 mmol/L (Ng et al. 2022). Preclinical studies indicate that FMT can restore microbial diversity and reduce systemic inflammation, leading to improvements in cardiovascular health. For example, FMT from healthy individuals to hypertensive patients showed a transient reduction in BP (Fan et al. 2022). In a randomized clinical trial (NCT04406129), hypertensive patients (*n* = 124) received FMT capsules shown to decrease in systolic BP after 1 week of FMT, but this difference did not maintain even after repeated interventions; however, it was notified that it was safe (Fan et al. 2025). Therefore, further detailed studies are required to establish the facts and also to advance the FMT‐based clinical interventions to treat CVDs.

In addition to FMT, postbiotics, nonviable microbial products such as SCFAs, enzymes, and peptides are being investigated for their cardiovascular benefits. They offer the advantage of stability and targeted delivery without the risks associated with live organisms (Rahimi et al. 2024). Regular physical activity is a natural and effective way to prevent or mitigate gut dysbiosis, which plays a key role in CVDs. Targeting the gut microbiome through exercise, alongside dietary interventions, may offer novel therapeutic approaches for CVD prevention and management (Yan et al. 2021).

Targeting the gut‐heart axis through probiotics, prebiotics, dietary interventions, pharmacological agents, and emerging strategies like FMT offers promising avenues for CVD management. These approaches highlight the potential of gut microbiota modulation in reducing systemic inflammation, improving lipid profiles, and enhancing overall cardiovascular health.

## 7 Future Directions and Challenges

Despite substantial progress in our understanding of gut microbiota, several questions remain, and future research must address these challenges to fully unravel the gut‐heart axis and translate findings into clinical practice. To understand how certain gut microbiota‐derived metabolites, such as TMAO, SCFAs, and LPS, affect cardiovascular health, advanced molecular and systems biology tools are necessary. Integrating multiomics approaches, such as metabolomics, proteomics, and transcriptomics, can lead to deeper understanding of these interactions. The gut‐heart axis describes the complex interplay between gut microbiota, their metabolites, and CVD risk and progression (Shariff et al. 2024). For example, elevated TMAO levels, resulting from microbial metabolism of dietary choline and carnitine, are strongly associated with increased risk of atherosclerosis and adverse cardiac events in both animal models (Zhen et al. 2023; Zheng and He 2022) and large‐scale human studies (Witkowski et al. 2020; Evans et al. 2023; Gatarek and Kaluzna‐Czaplinska 2021). Conversely, SCFAs generally exert protective effects by reducing inflammation and improving vascular function, although their impact can vary depending on the microbial composition and host factors (Shariff et al. 2024; Bui et al. 2023; Snelson et al. 2025). Although animal models are invaluable for investigating the role of gut microbiota, translating these findings to large‐scale human studies and clinical cohorts remains challenging due to factors such as individual variability, diet, lifestyle, and medication use.

Therefore, it is also crucial to understand the interindividual variation in gut microbiota composition and its interaction with host genetics. Personalized interventions, including diet modifications, prebiotics, probiotics, or postbiotics, could be developed to optimize gut microbiota for cardiovascular health. Research and clinical testing are required for novel therapeutic strategies, such as microbiota transplantation or small molecules targeting harmful metabolites. By investigation of microbiota‐drug interactions through pharmacomicrobiomics can enhance drug efficacy and minimize side effects in CVD therapies. The gut microbiome plays a vital role in both CVD risk and metabolism of xenobiotics which is a critical frontier for precision cardiology to develop personalized approaches. In fact, the microbiome is increasingly recognized as a minimal focused contributor on drug metabolism. More than 50 drugs are shown to metabolized by the gut microbiome (Haiser and Turnbaugh 2013). For example, Digoxin is a known medication to treat various heart conditions including atrial fibrillation and HF. A common anaerobe of the human colonic flora, Eubaterium lentum metabolizes digoxin to its reduced derivatives (Saha et al. 1983). Therefore, by elucidating these mechanisms and developing microbiota‐informed therapies, can drive a paradigm shift toward personalized cardiovascular care.

To establish relationships between changes in gut microbiota and CVD progression, large and longitudinal studies are necessary. These studies should investigate how gut and heart axis dynamics are influenced by lifestyle factors, aging, and comorbidities of CVD. Individual host factors such as genetics, diet, age, sex, and lifestyle significantly modulate the gut‐heart axis by influencing gut microbiota composition and the production of metabolites linked to cardiovascular health. These factors induce epigenetic changes impacting both heart and gut microbiome health. Several reports demonstrated that dietary factors could influence the epigenetic modifications that eventually elevate cardiovascular risk (Kalea et al. 2018; Kashtanova et al. 2016; Kiecolt‐Glaser et al. 2015). Chronic stress is also another factor that exacerbates gut dysbiosis and induces epigenetic changes that promote systemic inflammation, a key driver of heart disease (Akshay et al. 2023). Therefore, further exploration of the gut‐cardiac axis would pave a novel way for the development of new therapeutics for cardiovascular health management. The gut, brain, and heart have a bidirectional interaction, as suggested by emerging evidence. Understanding how the gut microbiota influences autonomic regulation and neurocardiac pathways could lead to new insights into CVD pathophysiology. The identification of universal biomarkers and therapeutic targets is challenging due to the immense diversity and variability of gut microbiota among individuals. Microbiome‐derived risk scores and diagnostic tools are at the forefront of translational microbiome research, offering new avenues for disease prediction, risk stratification, and noninvasive diagnostics. The new technological advances such as machine learning, single cell sequencing, approaches incorporating microbiome data have been shown to predict occurrence of HF. Recent studies have shown that gut microbiome composition varies significantly across the cardiovascular risk spectrum, as defined by conventional risk scores like Framingham (Versteylen et al. 2011). Specific bacterial species, such as Ruthenibacterium lactatiformans, *Flavonifractor plautii* , and *Collinsella stercoris* have high cardiovascular risk, while other bacterial species such as *Streptococcus thermophilus* are negatively regulated (Prins et al. 2024). Multivariable analyses demonstrated that cardiovascular risk profiles explain significant differences in the proportion of gut microbiome, and certain risk factors (diabetes, age, smoking, HDL) are linked to specific microbial patterns (Prins et al. 2024). Despite promising results, widespread clinical adoption of microbiome‐derived risk scores and diagnostics requires further validation, standardization of analytical methods, and robust calibration across populations around the globe.

Using well‐designed experimental models and human trials is important for validating hypotheses. Preclinical findings need to be translated into effective, scalable, and safe interventions for diverse populations, but it remains a significant hurdle. Microbiota‐based therapies and fecal microbiota transplantation raise ethical and regulatory concerns, requiring robust guidelines and monitoring frameworks. The gut microbiota represents a promising frontier in cardiovascular medicine. Addressing these future directions and challenges will enable the development of novel preventive, diagnostic, and therapeutic strategies, ultimately improving cardiovascular outcomes and patient care.

## Author Contributions

Narendra Kondapalli and Charles K. Thodeti conceived the project. Narendra Kondapalli, Venkatesh Katari, Kesha Dalal, Sailaja Paruchuri, and Charles K. Thodeti wrote and revised the manuscript.

## Conflicts of Interest

The authors declare no conflicts of interest.

## Data Availability

The authors have nothing to report.
